# Beyond policy narratives: exploring the role of pedagogical beliefs in classroom practices of secondary school Civics and Ethical Education teachers

**DOI:** 10.1186/s40561-021-00171-w

**Published:** 2021-10-30

**Authors:** Alemayehu Habte, Alemayehu Bishaw, Meskerem Lechissa

**Affiliations:** 1 Department of Pedagogy, Dilla College of Teacher Education, Dilla, Ethiopia; 2 College of Education and Behavioral Science, Department of Teacher Education and Curriculum Studies, Bahir Dar University, Bahir Dar, Ethiopia; 3 College of Education and Behavioral Science, Department of Teacher Education and Curriculum Studies, Bahir Dar University, Bahir Dar, Ethiopia

**Keywords:** Pedagogical beliefs, Classroom practice, Constructivism, Secondary school, Civics and ethical education, Ethiopia

## Abstract

In Ethiopia, secondary school Civics and Ethical Education has been offered to students with prime objective of producing competent and rational citizens. While policy narratives advocate constructivist pedagogy for achieving this goal of the curriculum, the reality on the ground hints that the subject is far behind achieving its stated goal. In line with this, teachers’ role in implementing the curriculum cannot be understated. Teachers are policy actors who implement the official curriculum. Their classroom practice; however, is largely dependent on their pedagogical beliefs. To this end, this study aimed at examining the role of secondary school Civics and Ethical Education teachers’ pedagogical beliefs in their perceived classroom practices vis-à-vis selected demographic variables. The study was conducted using correlational design participating 324 Civics and Ethical Education teachers from 43 government and private secondary schools in Addis Ababa city. Two-way multivariate analysis of variance and multiple regression were used to analyze the data. The regression analysis revealed that teachers' pedagogical beliefs explained 45.8% of the variance in classroom practice. Teachers were also found to have strong constructivist belief, even though they do not completely reject traditional belief per se. Their constructivist practice is; however, below the expected level, suggesting the interplay of contextual factor(s) which should be further studied. The findings implied the need to redefine continuous professional programs with emphasis on reflective teaching practice and improve climate of secondary schools.

## Introduction

Pedagogical belief is one of the most important aspects of teachers’ professional competence that affects teaching effectiveness (Thomas, Comfort, & Adams, 2013). The nature of teacher beliefs undoubtedly reflects the quality of instruction in classrooms. As a result, studying the relationship between teachers' beliefs and practices has become a vital component of educational research (Borg, 2015). As Ashton (2015) stated, research on teacher beliefs has expanded as “more researchers recognize that beliefs are a powerful influence on teachers’ thinking and behavior” (p. 43).

Teachers' pedagogical beliefs are a set of interconnected judgments about their classroom activity (Fives & Buehl, 2012). They are “subjective claims the individual [teacher] accepts as being true” (Buehl & Beck, 2015, p. 67) which dictate what the teacher says and does in the classroom (Farrell & Guz, 2019). Pedagogical beliefs, according to a large body of research, influence many elements of teachers' profession. They inform teachers' pedagogical decisions (Aksoy, 2015; Levin et al., 2013; Thibaut et al., 2018), instructional practices (Kim et al., 2013; Lebak, 2015; Wong & Luft, 2015), learning environment or classroom climate (Levin, 2015; Li, 2013; Rubie-Davies, 2015) and professional development (Buehl & Beck, 2015; Zhang & Liu, 2014). To put it in a nutshell, beliefs serve as "filters for interpretation, frames for defining problems, and guides or standards for action" (Fives & Buehl, 2012, p.478).

Accordingly, understanding the nature of teachers' pedagogical beliefs and how they connect to their practice is critical for improving teachers'  professional practice (Leem & Sung, 2019; Rodriguez & Magill, 2016); and to the success of educational reform initiatives (Fives & Buehl, 2016).  Ethiopia is currently undertaking a new educational reform known as 'The New Education Development Road map' (MoE, 2018). As the reform was primarily induced by the political change the country witnessed recently, the new government seems determined to heal the Achilles' heel of the old education system which was its failure to equip the youth with the required citizenship competences. Hence, we believe that it is a high time to examine Civics and Ethical teachers' pedagogical beliefs and practices so that policy makers can understand teachers' beliefs in order to amend, remodel, or reconstruct any policy that has direct repercussions in pedagogical practices (Karim et al., 2020).

### Context of the study

Most democratic, pluralistic countries are often challenged with the difficult task of offering citizenship education that can accommodate differences while also fostering the bonds, virtues, and practices that are necessary for the development of a socially cohesive democratic society (Banks, 2007; Dilworth, 2008). As a result, the production of good citizens, citizens who are well informed, concerned about the common good, and committed to democratic ideals, has become a top priority for many countries' educational systems (Birhanu, 2012).

In Ethiopian context, secondary school Civic and Ethical Education (hereafter CEE) aims to “provide the young with all the necessary capacities and skills, dispositions and attitudes; vision and meaning to life in general and to grasping of their specific manifestations as political, economic, social, and cultural phenomena” (Ministry of Education, 2009, p.31). The subject primarily aims at the development of critical and logical thinking, reasoning, judgment and decision making; as well as fostering positive attitudes and dispositions (MoE, 2009).

With these primary intentions, and as per the global and country-wide shift of education system towards constructivist paradigm, CEE was one of the secondary school subjects which witnessed several reforms (Yamada, 2014). The reforms mostly aimed to shift the teaching paradigm from transmission-based to inquiry-based orientation. Various policy documents, notably the 1994 Education and Training Policy (Federal Democratic Republic of Ethiopia, 1994), the Teacher Education System Overhaul (TESO) of 2003 (MoE, 2003), and the Secondary School Curriculum Framework of 2009 (MoE, 2009), have emphasized this transition. Consequently, secondary school teachers in general, and CEE teachers in particular, have battled to shift their classes away from traditional, teacher-centered context toward more constructivist environments.

Nonetheless, the initiative appears to fall far short of its objective. The few studies undertaken on Civics and Ethical Education at various levels of the educational system (e.g. Addis, 2013; Birhanu, 2012; Dawit, 2006; Endalkachew, 2016; Fetene, 2017; Girma, 2006; Gosa, 2018; Meron, 2006; Molalign, 2012; Mulugeta, 2009) reported either the subject's ineffectiveness in bringing about the anticipated changes in students' behavior or CEE teachers' incapacity to use proper instructional methods in their classrooms. Some even blamed it for the country's escalating ethnic tension which was partly caused by improper schooling. Officially acknowledging that CEE so far has not produced the anticipated results, a study commissioned by the Policy Study and Research Center (2017) ascribed the problem in part on teachers' incapability (Waltainfo, 2017). This issue was also recently confirmed by the government, which stated that the Education and Training Policy should be reviewed to ensure that it clearly articulates the balance between plurality and national unity in light of a new concept of being both a patriot and a nationalist (MoE, 2018).

Needless to say, this intention can only be realized with quality instruction (Mulugeta, 2009). Supporting this claim, several studies on citizenship education have pushed for constructivist pedagogy because these approaches are belied to enhance students' moral reasoning and civic awareness (Nucci, Creane, & Powers, 2015; Persson, 2015), tolerance (Maurissen, Bearber, & Claes, 2018), and civic engagement (Manganelli, Lucidi, & Alivernini, 2015; Quintelier & Hooghe, 2013). Thus, constructivist pedagogies are recommended in Civics and Ethical Education not only for their academic benefits, but also because they are the essential dispositions that effective citizens require in their daily lives. Simply put, citizenship education (Civics and Ethical Education) needs to include constructivist pedagogies as its important components if it is to produce democratic, critical citizens who actively and responsibly participate in society (Althof & Berkowitz, 2006; Haste, 2004).

### Theoretical framework

According to constructivist theories, learning is positioned in and shaped by socially, culturally, and historically significant contexts in which the learner and the environment negotiate authority, responsibility and tasks (Anderson & Stillman, 2013). Learning should serve learners' personal experiences because people develop knowledge and meaning via their own experiences (Riegler & Quale, 2010). Its proponents advocate that reasoning, critical thinking, knowledge construction and application, self-regulation, and mindful reflection are the goals of constructivist learning (Bailey & Colley, 2015). As a result, a constructivist approach to learning allows students to have tangible, contextually meaningful experiences in which they can self-organize, invent, discover patterns, be curious, raise questions and hypotheses, model, analyze, and support their views. Our research focuses on how teachers’ beliefs impact their perceived classroom practices. By implication, it also provides an important lens through which one may examine how students construct knowledge in secondary school CEE classrooms. Accordingly, we used constructivism as the primary theoretical framework.

### Statement of the problem

Several studies on citizenship education have pushed for constructivist pedagogy (e.g. Banks, 2008; Schuitema et al., 2009; Kaya, 2009). These approaches are believed to enhance students’ moral reasoning and civic awareness (Nucci et al. 2015; Persson, 2015), tolerance (Maurissen et al. 2018), and engagement in the society (Manganelli et al., 2015; Quintelier & Hooghe, 2013).

Nevertheless, CEE curriculum implementation is heavily reliant on the activities of teachers, who have their own set of firmly held beliefs (Fives & Buehl, 2012). Teachers provide learning opportunities that are consistent with their pedagogical views, and these opportunities have the potential to influence students' citizenship competence (Knowles, 2017; Knowles & Castro, 2019). Because Civics and Ethical Education is molded by a confluence of teachers' and students' views and identities (Epstein, 2001), analyzing these linkages has huge pedagogical implications for how it is taught in the classroom and how future teachers are prepared at the university.

However, despite their key role as frontline curriculum implementers with the moral and social imperative of preparing citizens (Mhlauli, 2011), teachers' practical expertise is often underestimated, and too little attention is placed on the criticality of CEE teachers' pedagogies (Jennings, 2003). Teachers' beliefs on civics (Citizenship) education, according to Reichert and Torney-Purta (2019), are relatively unknown. Other researchers also backed up this assertion by stating that there is a general dearth of empirical research and a need for additional in-depth studies in this field (e.g. Davies, 2000; Kerr, 2000).

Though there are several studies on teachers' beliefs and practices around the world, most of them have focused on either language (e.g., Li, 2013; Zhang & Li, 2014), mathematics and science (e.g., Lebak, 2015; Wong & Luft, 2015), or teachers' beliefs about technology integration (e.g., Kim et al., 2013). However, little is known about how teachers' beliefs are linked to civic and citizenship education instruction (Reichert, Lange & Chow, 2021). The situation is similar in Ethiopia, where there is a scarcity of empirical evidence (Semela, Bohl, & Kleinknecht, 2013). As far as we know, prior local studies (e.g. Endalkachew, 2016; Gosa, 2018; Semela et al., 2013; Yamada, 2014) never studied teachers' pedagogical beliefs and their relationship to classroom practices.

Furthermore, the nature of CEE and how it is implemented in classrooms differs amongst educational systems, schools, and teachers (Reichert et al., 2021). Nevertheless, the literature offers limited studies conducted on CEE teachers practice of constructivist pedagogy in relation to demographic characteristics and teachers’ pedagogical beliefs. Hence, we believed that it is imperative to assume that teachers’ classroom practices may differ according to their demographic characteristics such as gender, education level, teaching experience and school type. As a result, the purpose of this study was to examine the relationship between secondary school CEE teachers’ pedagogical beliefs and classroom practices *vis-à-vis* selected demographic variables in Addis Ababa city.

## Research questions

Specifically, this study was intended to answer the following research questions:

1. What are the pedagogical beliefs held by secondary school CEE teachers of Addis Ababa city?
2. Is there a statistically significant difference in CEE teachers' pedagogical beliefs and classroom practices based on gender, experience, educational qualifications, and school type?
3. Is there statistically significant relationship between CEE teachers’ pedagogical beliefs and classroom practices?
4. To what extent do CEE teachers’ pedagogical beliefs predict their classroom practices?

## Research design

In this study, a correlational design was adopted. A correlational design uses correlational statistics to find the direction and size of a relationship between variables without any manipulation (Creswell, 2012; Field, 2009). The correlational design was deemed to be compatible with the main goal of this study, which was to investigate the relationship between secondary school CEE teachers' beliefs and practices with regard to demographic variables such as gender, experience, education level, and school type.

### Sampling

324 CEE teachers from 43 government and private secondary schools in Addis Ababa city participated in the study. To produce samples that accurately represent the population under study, a multi-stage sampling technique was adopted. First, four sub-cities were picked from a total of 10 sub-cities using simple random sampling procedure. This first stage aided in the identification of research sub-cities (i.e. Kolfe Keranio, Nifas Silk, Kirkos and Yeka). Then, using stratified random sampling with proportional allocation, the number of schools to be taken from each sub-city was determined. The type of school ownership (public/private) was employed as stratum in this case. Finally, individual respondents (CEE teachers) from the schools identified in the previous stage were contacted.

### Data collection instruments

In this study, adapted versions of two instruments were used in the data collection process. The next section provides a quick overview of these measurement instruments.

#### Pedagogical beliefs

The Teaching and Learning Conceptions Questionnaire (TLCQ), developed by Chan and Elliot (2004) and further validated by Lee, Zhang, Song, and Huang (2013), was used to assess CEE teachers' pedagogical beliefs. The conceptions about teaching and learning refer to the beliefs held by teachers about their preferred ways of teaching and learning, hence pedagogical beliefs. The TLCQ had 30 items, representing two dimensions: Constructivist Conceptions (12 items), and Traditional Conceptions (18 items). The items were measured along a five-point likert scale, ranging from strongly disagree to strongly agree. Constructivist Conception included items such as “*Good CEE teachers always encourage students think for answers themselves*” and “*During CEE lessons, students should have ample opportunities to explore, discuss and express their ideas*”. On the other hand, traditional conception included items such as “*During CEE lesson, it is the good students who keep quiet and follow teacher’s instruction in class*” and “*Good CEE teaching occurs when there is mostly teacher talk in the classroom*”. The CTLQ has been used in a number of studies in Asia to investigate teachers' pedagogical beliefs (concepts of teaching and learning) (e.g., Chan and Elliott 2004; Chan, Tan & Khoo, 2007).

#### Teachers’ Pedagogical Practice

This was assessed using an adapted version of Taylor, Fraser, and Fisher's (1997) Constructivist Learning Environment Scale (CLES). The CLES was developed with a focus on the constructivist learning environment, allowing teachers to assess how well they implement constructivist ideas in their classrooms (Taylor et al., 1997). The CLES has been used and validated in many studies (e.g. Aldridge et al., 2000). The original CLES comprised of five scales (30 items) related to corresponding aspects of constructivism, namely; Personal Relevance (e.g. “*Students learn about the world outside of school*”), Uncertainty (e.g. “*Students learn that social realities (ideas) are influenced by people’s cultural values and opinions*”), Critical Voice (e.g. “*Students can express concern about anything that prevents them from learning*”), Shared Control (e.g. “*Students help me to decide how well they are learning*”) and Student Negotiation (e.g. “*Students can freely express their opinion, even when it was different from other students*”) (Taylor et al., 1997). CLES has a 5-point Likert-type frequency response scale which comprises the categories: Almost Always (5), Often (4), Sometimes (3) Seldom (2), and Almost Never (1). In this study, based on input from the pilot study and subsequent development by other researchers who used the instrument in their investigations, each sub-scale was reduced to five items. This was done by deleting negatively worded items and ones that appeared repetitive or confusing.

### Validation of instruments

The original questionnaires were translated into Amharic language by the researcher and reviewed by language experts. After translation, a pilot study was conducted to identify whether there were any sections that might be incomprehensible. The pilot study was conducted among 33 CEE teachers in six secondary schools. The questionnaire, along with an assessment tool, was delivered to participants, explaining the purpose of the pilot study and requesting them to assess the questionnaire and pinpoint areas that required improvement or clarification.

## Psychometric properties of CLES

The 25 items were subjected to Principal Component Analysis (PCA) using Varimax Rotation to determine the validity of the CLES. The Kaiser–Meyer–Olkin (KMO) test confirmed the analysis' sampling adequacy, with KMO = 0.914. The correlations between items were large enough for PCA, according to Bartlett's test of sphericity, x^2^ (300) = 5276.984, *p* = 001. The five-component solution explained 70.863% of the variance, with component 1 accounting for 36.062%, component 2 for 12.791%, component 3 for 9.124%, component 4 for 7.477%, and component 5 for 5.409%. The  five components had eigenvalues larger than 1, ranging from 1.352 to 9.015.

Cronbach alpha coefficients were used to check item reliability, and all individual items within scales, as well as the scales themselves, were found to be highly reliable with a score of above 0.70. A value higher than 0.7 is acceptable; however, values greater than 0.8 are preferred (Pallant, 2016). Cronbach alphas results for the five sub-scales were Critical voice (α = 0.89), Uncertainty (α = 0.89), Relevance (α = 0.87), Shared Control (α = 0.92), Negotiation (α = 0.88) and overall CLES (α = 0.92).

Then, both convergent and discriminant validity were tested. An Average Variance Extracted (AVE) of 0.50 or higher, or a Composite Reliability (CR) of 0.70 or higher, can be used as a good rule of thumb for convergent validity at the construct level (Collier, 2020; Hair et al., 2006). As presented in Table 1, all constructs demonstrated satisfactory convergent validity. Additionally, the Maximum Shared Squared Variance (MSV) for all constructs is less than AVE and the square root of AVE is higher than their correlation value, confirming discriminant validity. If MSV is less than AVE and Square root of AVE much more than inter-construct correlations then discriminant validity is established. Another way to show the evidence of discriminant validity is to use the average shared squared variance (ASV). Discriminant validity can be achieved when the AVE is greater than the ASV (Collier, 2020). The ASV was computed by averaging the inter-construct squared correlation. Table 1 shows that the AVE values of all factors are higher than the ASV which indicates discriminant validity.

**Table.1:** Construct reliability, convergent and discriminant validity results of the CFA model

	CR	AVE	MSV	ASV	MaxR(H)	1	2	3	4	5
Shared Control	0.919	0.694	0.271	0.358	0.932	**0.833**				
Critical Voice	0.800	0.572	0.135	0.410	0.801	0.314	**0.756**			
Uncertainty	0.898	0.640	0.200	0.428	0.932	0.355	0.618	**0.800**		
Relevance	0.878	0.591	0.200	0.361	0.881	0.290	0.386	0.424	**0.769**	
Negotiation	0.885	0.606	0.271	0.363	0.889	0.473	0.323	0.314	0.342	**0.778**

The diagonal values in bold are the square root of AVE while values below it are correlation between the respective constructs. CR = composite reliability; AVE = average variance extracted; MSV = maximum shared variance; ASV = average shared variance; MaxR(H) = maximum reliability

**Fig. 1:**
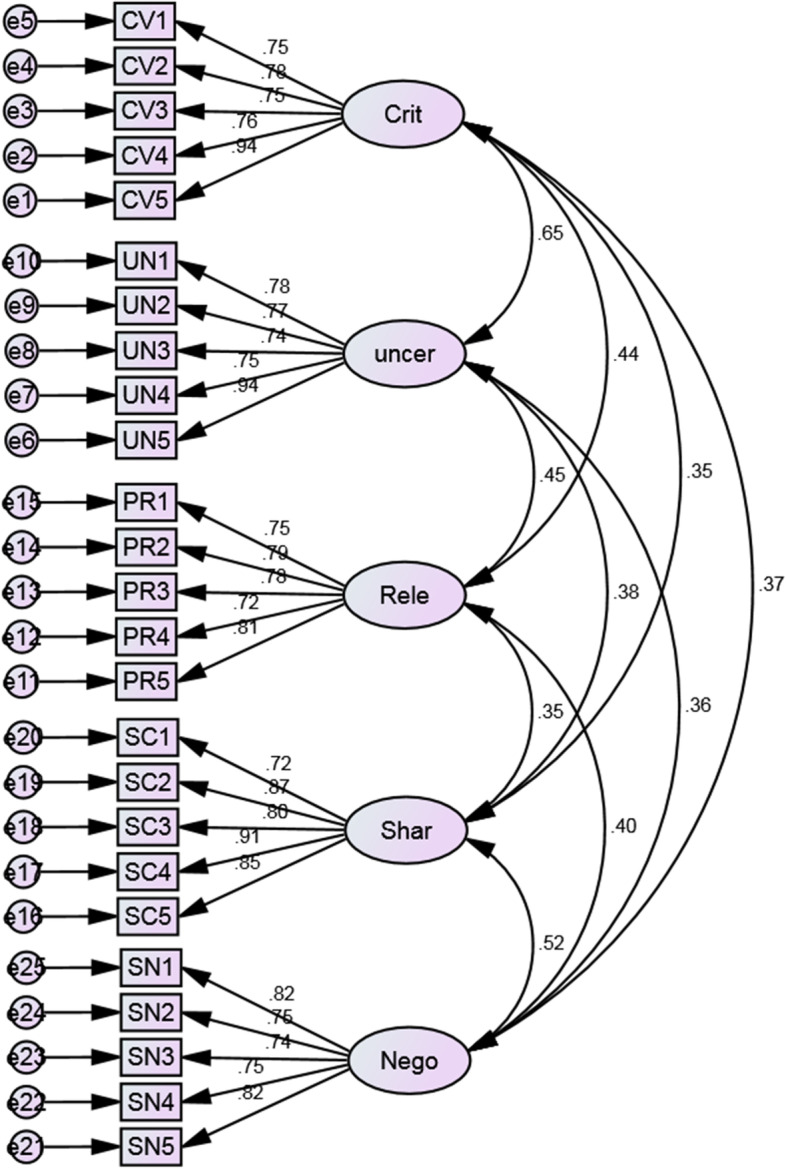
Path diagram of the five-factor model for the constructivist learning environment Scale (CLES)

Moreover, Confirmatory Factor Analysis (CFA) with AMOS 23 (Analysis of Moment Structures) software was conducted to further validate the instrument comprising the aforementioned five scales (see Fig. 1 below). The CFA results implied a model fit. The fit indices revealed χ2 = 401.610, *df* = 265, χ2/sd = 1.516; Goodness-of-fit index (GFI) = 0.910; Tucker Lewis Index (TLI) = 0.970; Comparative Fit Index (CFI) = 0.973; Standardized Root mean square residual (SRMR) = 0.0377; Root Mean-Square Error of Approximation (RMSEA) = 0.040; Adjusted Goodness-of-fit index (AGFI) = 0.889. Most researchers consider these values to be indicative of a good model fit (Brown, 2015; Collier, 2020; Hair et al., 2006; Kline, 2011). As a result, the instrument was confirmed as a valid and reliable measurement tool for measuring CEE teachers' constructivist classroom practice.

## Psychometric properties of CTLQ

The CTLQ has been used in a number of studies to investigate pre-service teachers' pedagogical beliefs (teaching and learning conception) in a number of Asian nations (e.g. Chan, 2004; Chan & Elliot, 2004; Chan et al., 2007). Nonetheless, the instrument's use to practicing teachers has been confined to few studies. The current study was one of those attempts to validate the instrument's usability for practicing teachers in Ethiopian context.

To test the validity of the TLCQ, the 30 items were subjected to Principal Component Analysis (PCA) using Varimax rotation. The Kaiser–Meyer–Olkin (KMO) test confirmed the analysis' sampling adequacy, with KMO = 0.965, well above the acceptable limit of 0.5. The correlations between items were large enough for factor analysis, according to Bartlett's test of sphericity, × 2 (435) = 5908.701, p = 0.001. Two factors similar to the original instrument had eigenvalues over 1 and together explained 56.757% of the total variance, with traditional belief contributing 39.727% and constructivist belief contributing 17.031%. The items grouped on the same factors as the original authors’, with factor 1 representing traditional conception and factor 2 reflecting constructivist conception having 18 and 12 items, respectively. The correlation between the two variables was *r* = -0.36, which supported  the original authors' suggestion that traditional belief items and constructivist belief items can  be used as independent scales (Chan & Elliot, 2004).

**Fig. 2:**
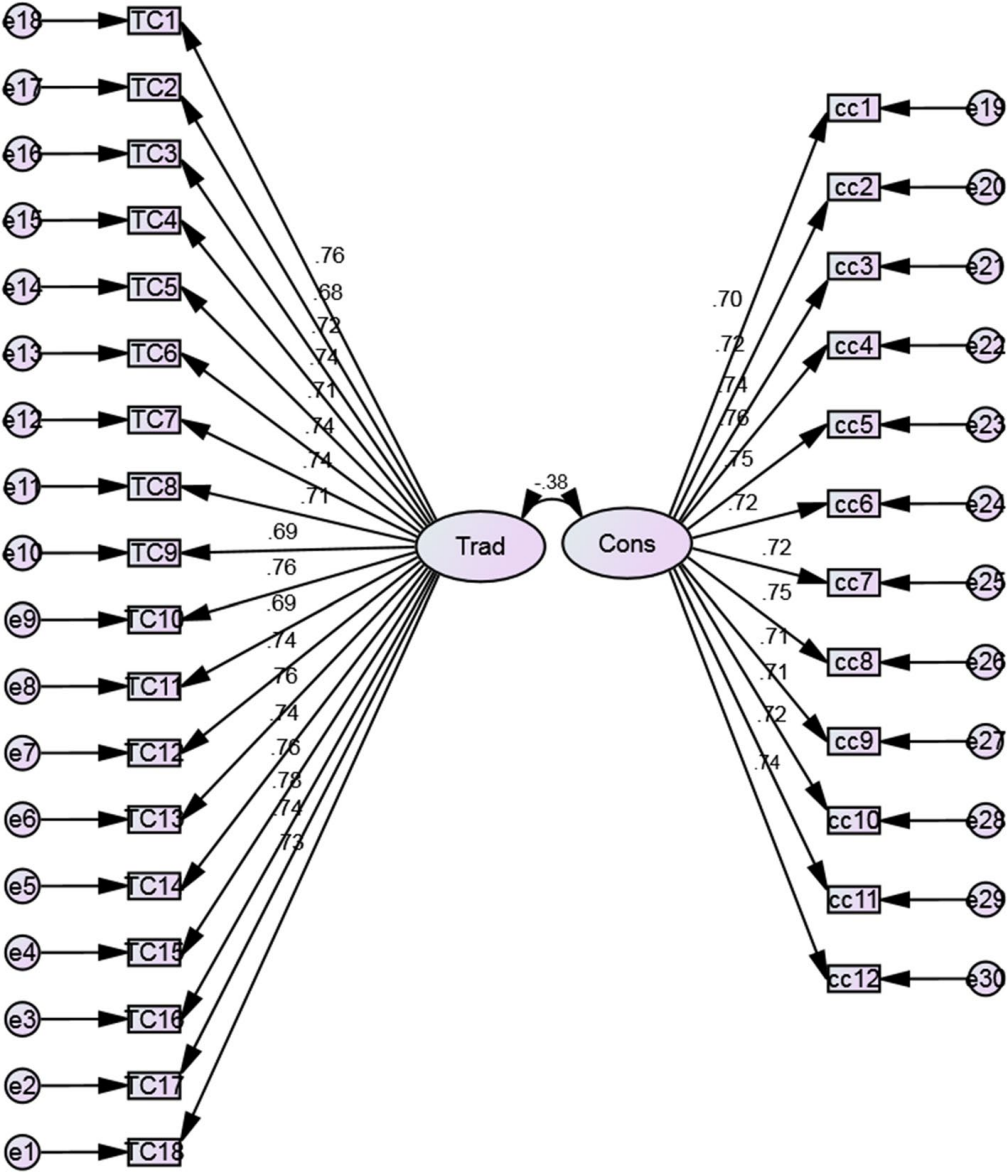
Path diagram of the two-factor model for the conception about teaching and learning questionnaire

Cronbach alphas were calculated for the two components, yielding the following results: traditional belief (18 items) α = 0.954 and constructivist belief (12 items) α = 0.931. According to Field (2009), a Cronbach alpha of 0.7 to 0.8 is sufficient for this type of test. Additionally, Confirmatory Factor Analysis (CFA) with AMOS 23 was conducted to further validate the instrument comprising the two scales (see Fig. 2). The CFA results implied a model fit. The fit indices revealed χ2 = 493.523, *df* = 404, χ2/sd = 1.222; GFI = 0.910; TLI = 0.983; CFI = 0.984; SRMR = 0.0367; RMSEA = 0.026; AGFI = 0.896. Most academics consider these values to be indicative of a strong model fit to the data (Collier, 2020; Hair et al., 2006; Kline, 2011). As a result, the instrument was confirmed as a valid and reliable measurement tool for measuring CEE teachers' pedagogical beliefs.

### Data analysis

Descriptive statistics like mean, standard deviation, and one-sample t-test were utilized in the first section of this investigation. The second section of the study employed a two-way MANOVA to investigate if there were any significant differences in teachers' beliefs and practices based on demographic characteristics. Before using two-way MANOVA, all assumptions including linearity, multivariate normality, and variance–covariance homogeneity were tested. Except for Levene's test of equality of error variances for one of the three variables (i.e. constructivist belief), the data met all of the assumptions. Pallant (2016) recommends using a more conservative alpha threshold for establishing significance for that variable in the univariate F-test if the data violates the equality of variances assumption. Similarly, Tabachnick and Fidell (2013) recommend an alpha of.025 or.01 instead of the conventional 0.05 level. As a result, during the Univariate F-test, an alpha value of.017 was used. Pillai's Trace was employed in this investigation because it is more reliable when assumptions are violated (Tabachnick & Fidell, 2013).

Pearson Product Moment Correlation was used to examine the relationship between teachers' beliefs and practices in the following stage. Finally, multiple-regression analysis was performed to see the degree to which teachers' pedagogical beliefs predict their classroom practices. The data's appropriateness was confirmed before the regression analysis. In this regard, the normality of the dependent variable's distribution (i.e. Constructivist Classroom Practice) was confirmed, and no outliers were found. Histograms were used to evaluate the actual shape of the distribution, which showed that the scores were reasonably normally distributed. An examination of the Normal Q-Q Plot also corroborated this.

Mahalanobis distances were used to test multivariate normality. Pallant (2016) claims that if the maximum value for Mahalanobis distance is smaller than the critical value, it is fair to believe that there were no significant multivariate outliers. In this study the maximum value for Mahalanobis distance (8.17) is pretty much less than the critical value (13.82). Multicollinearity was checked by running correlation to check the strength of the correlations among the independent variables. Correlations up around 0.8 or 0.9 are reason for concern (Pallant, 2016). In this study, the correlation between the two independent variables (i.e. traditional belief and constructivist belief) was, *r* = -0.36, which according to Pallant (2016) is not a concern. Tolerance, Variance Inflation Factor (VIF), and Condition Index (CI) were also looked into. The tolerance and VIF values for the two variables were found to be 0.87 and 1.15, respectively, with CI values of 1.00–20.89. The result revealed that there is no problem of multicollinearity between the independent variables because the VIF is less than 10 and the CI is likewise less than 30 (Tabachnick & Fidell, 2007). At the same time, considering the Durbin-Watson value (D-W = 1.94) it was confirmed that there was no autocorrelation (Tabachnick & Fidell, 2007).

## Results

The findings of the study are discussed below under four headings.

### Demographic characteristics of participants

The participants of the study consisted of teachers (n = 324) who were working in public and private secondary schools in Addis Ababa City. 38.9% (126) of these teachers were female while 61.1% (198) were male. In terms of professional experience, 26.2% (85) of the teachers who participated in the study had a teaching experience between 1–5 years, 34.6% (112) of them had 6–10 years of experience, 23.1% (75) of them had 11–15 years of experience, and 16% (52) of them had professional experience of 16 years and more. Regarding the type of school (Government versus Private) they work in, 61.4% (199) of them were from Government secondary schools while 38.6% of them (125) were employed in private secondary schools. In addition, it was found that 27.2% (88) of them had Master of Arts/Masters of Education (MA/M.Ed.) degree, while 72.8% (236) of them had Bachelor of Arts/Bachelor of Education (BA/B.Ed.) degree.

### Teachers’ pedagogical beliefs and constructivist practices

As can be seen in Table 1 below, teachers’ mean score for both constructivist belief and traditional belief are above the hypothesized mean of 3 and statistically significant. Teachers reported higher levels of constructive belief (*M* = 3.91*, SD* = *0.5*9) than the hypothesized mean of 3, *t* (323) = 27.86, *p* < 0.001. Similarly, teachers’ score on traditional dimension (*M* = 3.12*, SD* = 0.76) was also above the average point and statistically significant, *t* (323) = 2.94, *p* = 0.004.

On the contrary, teachers’ overall constructivist classroom practice as measured by the CLES was *M* = 2.91, *SD* = 0.61, which is significantly below the hypothesized mean of 3.0,* t* (323) = -2.59, *p* = 0.01. When each dimensions of CLES were considered, teachers reported practices indicated that their scores on the four dimensions of CLES i.e. “Critical Voice” (*M* = 2.82, *SD* = 0.86), “Uncertainty” (*M* = 2.86, *SD* = 0.82), “Personal Relevance” (*M* = 2.86, *SD* = 0.91), and “Student Negotiation” (*M* = 2.96, *SD* = 0.86) were found to be below the hypothesized mean of 3.0. Only the “Shared Control” dimension was minimally above the hypothesized mean score (*M* = 3.07, *SD* = 0.85), though the mean difference was not statistically significant *(t* (323) = 1.51, *p* = 0.132). The results for “Critical Voice” *(t* (323) = -3.80, *p* < 0.001), “Uncertainty” *(t* (323) = -3.13, *p* = 0.002), and “Relevance” *(t* (323) = -2.81, *p* = 0.005) were significantly below hypothesized mean of 3.0. The mean difference for “Student Negotiation” dimension was not found statistically significant *(t* (323) = -0.92, *p* = 0.358) (see Table 2).

**Table.2:** Mean, standard deviation and one sample t-test results of variables

	M	SD	*df*	*t*	*p*
Traditional belief	3.12	.76	323	2.94	.004
Constructivist belief	3.91	.59	323	27.86	.000
Overall constructivist practice	2.91	.61	323	-2.59	.010
Critical Voice	2.82	.86	323	-3.80	.000
Uncertainty	2.86	.82	323	-3.13	.002
Personal Relevance	2.86	.91	323	-2.81	.005
Shared control	3.07	.85	323	1.51	.132
Student Negotiation	2.96	.86	323	-.92	.358

*M* = Mean, *SD* = Standard deviation, df = degree of freedom

### Comparisons of pedagogical beliefs and practices by gender and teaching experience

A two-way multivariate analysis of variance (MANOVA) was conducted to determine whether teachers’ score on traditional belief, constructivist belief and constructivist practice significantly differ according to teachers’ gender, teaching experience and their interaction. Using Pillai’s Trace, the combined dependent variables were significantly different according to gender (Pillai’s V = 0.038, F (3, 314) = 4.16, *p* < 0.001, multivariate Ƞ^2 =^0.038) and teaching experience (Pillai’s trace V = 0.123, F (9,948) = 4.49, *p* < 0.001, multivariate Ƞ^2^ = 0.041). However, the interaction between gender and teaching experience was not significant, Pillai’s trace V = 0.023, F (9,948) = 0.82, *p* = 0.602, multivariate Ƞ^2^ = 0.008 (see Table 3).

**Table.3:** Multivariate tests of gender and teaching experience

Effect		Value	F	Hypothesis df	Error df	Sig	Partial eta squared
Intercept	Pillai's Trace	.993	14,341.598	3.000	314.000	0.000	.993
Gender	Pillai's Trace	.038	4.158	3.000	314.000	.007	.038
Experience	Pillai's Trace	.123	4.490	9.000	948.000	.000	.041
Gender* Experience	Pillai's Trace	.023	.815	9.000	948.000	.602	.008

To evaluate the impact of each effect on the individual DVs, a Univariate F-test using a new alpha level of 0.017 and Scheffe Post hoc analysis were conducted as follow-up tests. The result indicated that traditional belief score significantly differs in terms of gender. Female teachers traditional belief score (*M* = 3.29, *SD* = 0.76) and male teachers’ traditional belief score (*M* = 3.02, *SD* = 0.74) differed significantly, (F (1,316) = 9.92, *p* < 0.001, Ƞ^2^ = 0.030). Though male teachers’ mean score on constructivist belief (*M* = 4.01, *SD* = 0.50) is higher than their counterparts (*M* = 3.59, *SD* = 0.60), the difference was not found statistically significant using the new alpha value of 0.017 (F (1,316) = 5.19, *p* = 0.023, Ƞ^2^ = 0.016). Similarly, though male teachers’ score on constructivist practice (*M* = 2.96, *SD* = 0.58) is slightly higher than female teachers’ score (*M* = 2.84, *SD* = 0.64), the difference was not statistically significant (F (1,316) = 2.93, *p* = 0.088, Ƞ^2^ = 0.009).

Both traditional belief scores (F (3,316) = 8.58, *p* < 0.001, Ƞ^2^ = 0.075) and constructivist belief scores (F (3,316) = 8.48, *p* < 0.001, Ƞ^2^ = 0.074) significantly differed based on teaching experience. Scheffe’ post hoc results for teaching experience and traditional belief indicated that mean scores of novice teachers (with experience of 5 years or less) (*M* = 2.79, *SD* = 0.79) significantly differed from teachers with experience category of 6 to 10 years (*M* = 3.17, *SD* = 0.71), 11 to 15 years (*M* = 3.26, *SD* = 0.67) and with teachers having 16 years or more experience (*M* = 3.38, *SD* = 0.75). Novice teachers also significantly differed in their constructivist belief mean score (*M* = 4.15, *SD* = 0.57) with teachers with experience category of 11 to 15 years (*M* = 3.73, *SD* = 0.59) and with teachers having 16 years or more experience (*M* = 3.70, *SD* = 0.47); but not with teachers with experience category of 6 to 10 years (*M* = 3.94, *SD* = 0.58). On the other hand, the univariate F-test indicated that constructivist practice did not differ for gender (F (1,316) = 2.93, *p* = 0.088, Ƞ^2^ = 0.009), teaching experience (F (3,316) = 2.57, *p* = 0.055, Ƞ^2^ = 0.024) as well as the interaction of gender and teaching experience (F (3,316) = 0.48, *p* = 0.700, Ƞ^2^ = 0.004) (see Table 4).

**Table.4:** Results of test of between subject effects based on gender and teaching experience

Source		Type III sum of squares	Df	Mean square	F	Sig	Partial eta squared
Corrected model	Traditional	21.651^a^	7	3.093	5.986	.000	.117
	Constructivist	13.301^b^	7	1.900	6.133	.000	.120
	Practice	5.102^c^	7	.729	1.995	.055	.042
Intercept	Traditional	2837.034	1	2837.034	5490.481	.000	.946
	Constructivist	4207.254	1	4207.254	13,579.402	.000	.977
	Practice	2357.019	1	2357.019	6452.324	.000	.953
Gender	Traditional	5.124	1	5.124	9.916	.002	.030
	Constructivist	1.609	1	1.609	5.194	.023	.016
	Practice	1.070	1	1.070	2.928	.088	.009
Experience	Traditional	13.305	3	4.435	8.583	.000	.075
	constructivist	7.880	3	2.627	8.478	.000	.074
	Practice	2.811	3	.937	2.565	.055	.024
Gender * experience	Traditional	1.207	3	.402	.778	.507	.007
	Constructivist	1.330	3	.443	1.431	.234	.013
	Practice	.520	3	.173	.475	.700	.004
Error	Traditional	163.283	316	.517			
	Constructivist	97.905	316	.310			
	Practice	115.434	316	.365			
Total	Traditional	3346.219	324				
	Constructivist	5059.938	324				
	Practice	2868.392	324				
Corrected total	Traditional	184.934	323				
	Constructivist	111.206	323				
	Practice	120.536	323				

^a^R Squared = .117 (Adjusted R Squared = .098)

^b^R Squared = .120 (Adjusted R Squared = .100)

^c^R Squared = .042 (Adjusted R Squared = .021)

### Comparisons of pedagogical beliefs and practices by educational level and school type

A two-way multivariate analysis of variance was conducted to determine whether teachers with BA/B.Ed. and those with MA/M.Ed. working in Private and Government secondary schools significantly differ in their traditional belief, constructivist belief, and constructivist practice mean score. Furthermore, analysis was made to test whether there was significant interaction between qualification and school type on the three dependent variables. The results showed that the main effect for educational qualification was significant, Pillai’s trace V = 0.047, F (3,318) = 5.19, *p* = 0.002, multivariate Ƞ^2 =^0.047. The main effect of school type was also significant, Pillai’s trace V = 0.025, F (3,318) = 2.67, *p* = 0.048, multivariate Ƞ^2^ = 0.025. However, the interaction between educational qualification and school type was not significant, Pillai’s trace V = 0.019, F (3,318) = 2.01, *p* = 0.112, multivariate Ƞ^2^ = 0.019 (Table 5).

**Table.5:** Multivariate tests of education level and school type

Effect		Value	F	Hypothesis df	Error df	Sig	Partial eta squared
Intercept	Pillai's trace	.992	12,787.429	3	318	0.000	.992
Education	Pillai's trace	.047	5.192	3	318	.002	.047
School type	Pillai's trace	.025	2.668	3	318	.048	.025
Education * school type	Pillai's trace	.019	2.010	3	318	.112	.019

Univariate F-test analysis using the new alpha value of 0.017 indicated that constructivist belief significantly differed in terms of educational qualifications (F (1,320) = 13.60, *p* < 0.001, Ƞ^2^ = 0.041). Traditional belief also significantly differed based on educational qualification (F (1,320) = 6.00, *p* = 0.015, Ƞ^2^ = 0.018). Mean score of teachers with graduate degrees (*M* = 2.93, *SD* = 0.72), was lower than teachers with undergraduate degrees (*M* = 3.19, *SD* = 0.76) on traditional belief. On the other hand, teachers with graduate degrees were found to hold a stronger constructivist belief (*M* = 4.11, *SD* = 0.56), than their counterparts (*M* = 3.83, *SD* = 0.58). Nevertheless, constructivist classroom practice did not differ in terms educational qualifications (F (1,320) = 2.93, *p* = 0.088, Ƞ^2^ = 0.009).

Descriptive statistics showed that traditional belief mean score of private school teachers (*M* = 3.01, *SD* = 0.74) was lower than mean score of teachers working in government schools (*M* = 3.19, *SD* = 0.76). Conversely, their score on constructivist belief dimension (*M* = 4.01, *SD* = 0.56) and constructivist practice dimensions (*M* = 2.96, *SD* = 0.65) were higher than the constructivist belief mean score (*M* = 3.84, *SD* = 0.60) and constructivist practice (*M* = 2.88, *SD* = 0.58) mean score of teachers working in government schools. Nevertheless, Univariate F-test analysis using the new alpha value of 0.017 indicated that constructivist belief (F (1,320) = 5.60, *p* = 0.019, Ƞ^2^ = 0.017), traditional belief (F (1,320) = 1.62, *p* = 0.204, Ƞ^2^ = 0.005) and constructivist practice (F (1,320) = 0.018, *p* = 0.893, Ƞ^2^ = 0.0) did not significantly differ in terms of school type (see Table 6).

**Table.6:** Results of test of between subject effects according to education level and school type

Source	Dependent variable	Type III sum of squares	Df	Mean square	F	Sig	Partial eta squared
Corrected model	Traditional	6.557^a^	3	2.186	3.921	.009	.035
	Constructivist	6.791^b^	3	2.264	6.937	.000	.061
	Practice	2.712^c^	3	.904	2.455	.063	.022
Intercept	Traditional	2317.176	1	2317.176	4156.920	.000	.929
	Constructivist	3950.250	1	3950.250	12,106.315	.000	.974
	Practice	2179.725	1	2179.725	5919.970	.000	.949
Education	Traditional	3.344	1	3.344	5.999	.015	.018
	Constructivist	4.438	1	4.438	13.601	.000	.041
	Practice	1.078	1	1.078	2.927	.088	.009
School type	Traditional	.903	1	.903	1.619	.204	.005
	Constructivist	1.828	1	1.828	5.601	.019	.017
	Practice	.007	1	.007	.018	.893	.000
Education* school type	Traditional	.393	1	.393	.705	.402	.002
	Constructivist	.382	1	.382	1.170	.280	.004
	Practice	.929	1	.929	2.522	.113	.008
Error	Traditional	178.376	320	.557			
	Constructivist	104.415	320	.326			
	Practice	117.824	320	.368			
Total	Traditional	3346.219	324				
	Constructivist	5059.938	324				
	Practice	2868.392	324				
Corrected Total	Traditional	184.934	323				
	Constructivist	111.206	323				
	Practice	120.536	323				

^a^R Squared = .035 (Adjusted R Squared = .026)

^b^R Squared = .061 (Adjusted R Squared = .052)

^c^R Squared = .022 (Adjusted R Squared = .013)

### Relationship between belief and practice

Pearson correlation coefficient was computed to see if there are statistically significant relationship between dimensions of teachers’ pedagogical beliefs (constructivist belief versus traditional belief) and overall practice and each dimensions of constructivist practice. The results indicated that there was a strong, negative correlation between traditional belief and constructivist classroom practice, *r* = –0.65, n = 324, *p* < 0.001. On the other hand, a negative moderate relationship between teachers’ traditional beliefs and four of constructivist practice dimensions i.e. critical Voice (*r* = -0.38, *p* < 0.01), Uncertainty (*r* = -0.39, *p* < 0.01), Personal Relevance (*r* = -0.39, *p* < 0.01), Shared Control (*r* = -0.46, *p* < 0.01) were identified. A strong negative relationship was identified between traditional belief and Student Negotiation (*r* = -0.70, *p* < 0.01) (see Table 7).

**Table.7:** Correlations between pedagogical beliefs and constructivist practice dimensions

	1	2	3	4	5	6	7	8
Traditional	**–**							
Constructivist	− .36^**^	**–**						
Critical	− .38^**^	.32^**^	**–**					
Uncertainty	− .39^**^	.28^**^	.62^**^	**–**				
Relevance	− .39^**^	.35^**^	.39^**^	.42^**^	**–**			
Shared	− .46^**^	.26^**^	.31^**^	.36^**^	.29^**^	**–**		
Negotiation	− .70^**^	.24^**^	.32^**^	.31^**^	.34^**^	.47^**^	**–**	
Practice	− .65^**^	.41^**^	.74^**^	.76^**^	.70^**^	.68^**^	.69^**^	**–**

^**^. Correlation is significant at the 0.01 level (2-tailed)

On the other hand, a significant positive, yet moderate relationship was observed between teachers’ constructivist belief and overall constructivist practice (*r* = 0.41, *p* < 0.01). The result also showed that while there was a moderate relationship of constructivist belief with two dimensions of constructivist practice i.e.with Critical voice (*r* = 0.32, *p* < 0.01) and Relevance (*r* = 0.35, *p* < 0.01); a weak relationship was evidenced with the other three dimensions i.e. Uncertainty (*r* = 0.28, *p* < 0.01), Shared Control (*r* = 0.26, *p* < 0.01) and Negotiation dimensions (*r* = 0.24, *p* < 0.01). In addition to this, it was found that there was a significant negative relationship between teachers’ traditional belief and constructivist belief scores (r = -0.36, p < 0.01).

### Predictors of teachers’ constructivist classroom practice

Finally, multiple regression analysis was computed to identify the degree to which teachers’ constructivist practices are explained by their pedagogical beliefs. Using the enter method, it was found that teachers’ pedagogical beliefs explain a significant amount of variance in teachers’ classroom practices. The results indicated that the model was a significant predictor of the dependent variable, F (2, 321) = 135.895, *p* < 0.01) (see Table 8).

**Table.8:** Multiple regression analysis for predicting teachers’ constructive classroom practice

Model	Unstandardized coefficients	Standardized coefficients		t	Sig	Correlations	
	B	SE	Beta(β)			Partial	Part
(Constant)	3.540	.243	14.538	.000			
Traditional Belief	– .466	.036	– .577	– 13.110	.000	– .520	– .486
Constructivist Belief	.212	.046	.203	4.619	.000	.293	.244

N = 324; R = .677; R Square = .458; Adjusted R^2^ = .455

As clearly shown in Table 8, the 2 independent variables were found to be significant predictors of teachers’ classroom practices. Traditional belief was found to negatively contribute to constructivist classroom practices (β = -0.577, t = -13.110, *p* < 0.01), while Constructivist belief positively predicted constructivist classroom practices (β = 0.203, t = 4.619, *p* < 0.01). The results also indicated that the two variables together, as expressed in the R-square, explained  45.8 per cent of the total variance in teachers’ constructivist classroom practices (R= 67.7; R^2^ = 45.8). Of these two variables, traditional belief makes the largest unique contribution (β = –0.577), although constructivist belief also made a statistically significant contribution (β = 0.203).

When the coefficients of the Part correlation are considered, if we square this value, we get an indication of the contribution of that variable to the total R square. In other words, it tells us how much of the total variance in the dependent variable is uniquely explained by that variable and how much R square would drop if it wasn’t included in the model. In the above table, the traditional belief has a part correlation co-efficient of –0.486. When this result are squared we get 0.24, indicating that the variable explains 24 per cent of the variance in teachers’ classroom practices. For the constructivist belief the value is –0.244, which squared gives us 0.06, indicating a unique contribution of 6 per cent to the explanation of variance in classroom practices.

## Discussion

The main objective of this study was to examine the relationship between secondary school CEE teachers’ pedagogical beliefs and classroom practices along with selected demographic variables. To this end, we framed four research questions so as to achieve the main purpose of the study. What follows is a discussion of the results of the four research questions.

The first research question was intended to identify the pedagogical beliefs held by CEE teachers. Teachers in this study reported having a strong constructivist belief. However, the teachers' mean score on traditional belief, which is somewhat higher than the hypothesized mean of 3.0, indicates that they do not appear to completely reject traditional belief. This result was similar to prior studies (e.g., Baş & Entürk, 2019; Berger et al., 2018; Cheng et al., 2009, Sing & Khine, 2008), who found that participants in their studies were more predisposed to constructivist beliefs.

Regarding constructivist practice, it was found that teachers’ reponses fall on ‘sometimes’ category, which reveals that their implementation of constructivist pedagogy is below the expected level. This is somewhat different from the result of Wang (2016) who reported that teachers in their study reported relatively high level of constructivist learning environment in their classes.

The second research question intended to examine whether there is statistically significant difference in CEE teachers’ beliefs and practices according to gender, experience, qualification and school type. The results showed that while there was no statistically significant difference in constructivist belief among teachers by gender variable, there was a statistically significant difference in traditional belief. In this regard, female teachers were found to have a higher traditional belief than male teachers. The finding backed up the OECD's (2009) conclusion that female teachers  are more likely than male teachers to perceive instruction as direct knowledge transmission. Nonetheless, it  contrasted Lee et al's (2013) findings, which reported female teachers were more likely to hold a constructivist believe than a traditional belief. In terms of practice, we found that teachers’ adoption level of constructivist pedagogy is not significant in terms of gender. This corroborates findings of prior studies (e.g., Aliusta, Özer & Kan, 2015; Arseven, Sahin, & Kilic, 2016) which indicated that teachers’ implementation of student centered teaching is not significant in terms of gender.

In our study, a significant difference was found in terms of both traditional belief and constructivist belief based on teaching experience. Teachers with teaching experience of 1–5 years exhibited a higher constructivist belief than teachers with experience of 16 years and above. This result concurs with some prior studies (e.g., Baş & Şentürk, 2019; Şentürk & Zeybek, 2019) who found that more experienced teachers held more traditional belief than less experienced teachers but contrasts with the findings of Berger et al (2018) and OECD (2009) which reported that the more experience teachers had, the more they believed in constructivism and the less they believed in direct transmission.

With regard to teaching experience and  constructivist practice, we found that teaching experience had no significant effect on constructivist practice. This result happened to differ from other studies (e.g., Wang, 2016) who reported that teachers’ constructivist practice was related to their teaching experience to some extent. On the other hand, it corroborates with the study of Jones and Leagon (2014) who affirmed years of teaching experience is not significantly related to teacher effectiveness.

In our study, the result suggests that novice teachers have a higher level of constructivist belief. This is perhaps unsurprising, given that Teacher Education Institutes in Ethiopia have been reorganized in accordance with the constructivist paradigm and have been training teachers in this manner for almost two decades. As a result, the finding that young teachers with few years of experience exhibit greater constructivist beliefs than more experienced teachers might be viewed as a reflection of the pre-service training they got.

On the other hand, the shift in constructivist ideology among teachers from a higher to a lower score as their teaching experience increases could indicate two things. First, despite the on-the-job trainings provided to reorient teachers’ beliefs and practices, older teachers were more devoted to their previous techniques of "talk and chalk" teaching. This is consistent with what academics (e.g., Skott, 2015) refer to as the change-resistant nature of teacher belief. According to Pajares (1992), the sooner a belief is absorbed into the beliefs system, the longer it is employed, and thus the stronger and more commanding it becomes to the person who holds it. In the present study’s context, traditional beliefs about how people learn or how people teach others were incorporated into CEE teachers’ beliefs structure early during childhood and early schooling. Teachers, on the other hand, learn about constructivism pedagogy later in their careers, at initial or in-service teacher training. Traditional beliefs are; therefore, basic beliefs and naturally significant to CEE teachers since they have grown up with and used them regularly in previous cognitive processes (Hutner & Markman, 2016). Mansour (2013) confirmed that the belief-practice alignment was closer when teachers held more traditional beliefs and more divergent when they held constructivist belief.

Second, the fact that teachers begin their teaching careers with a more constructivist approach and then revert to traditional teaching as their experience grows might suggest a significant school contextual element is at work. This appears to be especially true in Ethiopia, where CEE is heavily regulated and regarded a politicized matter. As a result, even though CEE teachers advocate constructivism and are committed to put it into practice, factors such as school ethos, curriculum, exams and timetables may be found unaccommodating. The hidden curriculum; therefore, leaves no choice to teachers except to retreat to their core belief i.e. traditional beliefs. To put it another way, teachers may find traditional teaching more practical under present school systems in an unsupportive school climate (Hutner & Markman, 2016).

In terms of education level, teachers with graduate degrees were found to be more constructivists and less traditional in their pedagogical beliefs than teachers with BA/B.Ed. degrees. This finding is consistent with the findings of Lee et al (2013) and Şentürk and Zeybek (2019), who found that teachers with an MA/M.Ed. degree exhibit greater constructivist beliefs than teachers with merely a bachelor's degree. Nevertheless, constructivist classroom practice did not significantly differ in terms educational qualifications and school type. Similarly, though male teachers’ score on constructivist practice is slightly higher than female teachers’ score, the difference was not statistically significant. The result was similar with teaching experience, where the univariate F-test indicated there is no statistically significant difference in teachers’ constructivist practice according to teaching experience.

The third research question was intended to see if there exists statistically significant relationship between CEE teachers’ pedagogical beliefs and classroom practices. The results indicated that there was a strong negative correlation between traditional belief and overall constructivist classroom practice. On the other hand, a significant positive, yet moderate relationship was observed between teachers’ constructivist belief and overall constructivist practice. Interestingly, teachers reported a strong constructivist belief. Nonetheless, their constructive practice did not match up with their espoused belief. Teachers’ overall constructivist classroom practice was found below the expected level, and only a moderate relationship was found between constructivist belief and constructivist practice. Conversely, a strong negative relationship was found between traditional belief and constructivist practice. The result partly suggests that teachers’ reported belief was inconsistent with their actual practice. This finding agrees with several prior studies (e.g., Farrell & Vos, 2018; Guerra & Wubbena, 2017; Karim et al., 2020) which reported that teachers’ reflected either ineffective implementation or no evidence of implementation of their professed beliefs.

According to some studies (e.g. Borg, 2018; Buehl & Beck, 2015; Phipps & Borg, 2009), this belief-practice incongruity could be the result of a complex set of personal and contextual circumstances that limit teachers' ability to pay attention to their beliefs and teach in accordance with their stated beliefs. These factors, among others, may include social, institutional and classroom context, time constraints, prescribed curriculum, high-stakes examinations, situational constraints, school and district policies and school culture (Basturkmen, 2012; Phipps & Borg, 2009). Mansour (2013) in particular claimed that the social norms of the school community influence how teachers believe their enacted practices will be perceived. For CEE teachers, this tension between beliefs and practices occurs every class hour since most of the issues are prone to differing, often times controversial views.

The fourth research question was targeted at determining the extent CEE teachers’ pedagogical beliefs predict their classroom practices. The results of the regression analysis revealed that teachers' pedagogical beliefs significantly predicted their classroom practices, with the two dimensions of pedagogical belief accounting for 45.8% of the variances in classroom practice, with a traditional belief solely accounting for 24% of the variances. The result agrees with prior studies (such as, Berger et al., 2018; Farrell & Ives, 2015; Farrell & Yang, 2017; Thibaut et al., 2018) which reported strong correspondence between teachers’ beliefs and practices. In light of this finding, it can be stated that CEE teachers’ pedagogical beliefs is a significant predictor of their classroom practices in this study.

## Conclusion and recommendation

Secondary school Civics and Ethical Education classroom is an ideal platform from which students can establish a foundation of critical thinking, spirit of critical inquiry, problem solving, decision making, civic knowledge, tolerance, civic mindedness and other crucial civic skills and dispositions. To this end, CEE teachers need to create constructivist learning environment whereby students are exposed with multiple viewpoints, share their experiences, defend their viewpoints and learn intercultural tolerance which will eventually help them to effectively deal with the challenges in our contemporary society (Kahne & Westheimer, 2006).

Despite the fact that teachers’ constructivist belief score is higher than the traditional dimension score, the traditional belief score is somewhat higher than the hypothesized mean value indicates that teachers still have a traditional orientation. The fact that these teachers do not reject or agree with traditional beliefs does not necessarily imply that they ignore them. Hence, the result should be interpreted cautiously especially when the effect of traditional belief in the regression analysis is considered. It could mean that CEE teachers are either undecided in their beliefs, or hold layered belief systems with both traditional or constructivist beliefs coexisting in their belief systems (Fives, Lacatena, & Gerard, 2015; Zhang & Liu, 2014).

The current study also evidenced that there is a disconnection between teachers’ constructivist belief and their practices in secondary school CEE classroom. Teachers' beliefs and practices, according to a number of studies (e.g., Buehl & Beck, 2015; Levin, 2015; Pajares, 1992), are always situated in a physical setting in which constraints, opportunities, or external influences may come from sources at various levels, such as the individual classroom, the principal, the school, the curriculum, or the community, and bureaucratic influences. Classroom practice and beliefs become consistent when these external and internal factors match teachers' beliefs (Mansour, 2013). Conversely, when these circumstances get in the way of teachers'  convictions, classroom practice and beliefs become incongruent. Given their context, teachers may modify their beliefs to better fit their experience (Fives & Buehl, 2016). Accordingly, complete understanding of secondary school CEE classroom practices is only possible with a thorough study of the contextual constraints and opportunities that impact them. Therefore, initiatives to change teachers’ pedagogical practices must focus on teachers’ beliefs and the prevailing school culture and hidden curriculum that inevitably shape their classroom practice. Based on the results of the study the following recommendations were forwarded:

- Teachers who work in schools with an innovative school culture and a supportive administration are more likely to hold constructivist beliefs because these factors promote a change-friendly environment (Zhang & Liu, 2014). Accordingly, school principals must create friendly, positive school climate where teachers collaborate among themselves.
- Teachers need to be reflective of the impact of their own personal beliefs on their classroom practice (Mansy, 2014; Wachob, 2012). When teachers are aware of the impact their beliefs have on students’ learning, they are more likely to use more effective teaching approaches. Through reflection teachers can realize how their ideas can help or obstruct good classroom activities by acknowledging their existing views (Fives, et al., 2015). Hence, the Ministry of Education of Ethiopia needs to earnestly consider CEE teachers’ beliefs in the currently developing ‘The New Education Development Roadmap’ so as to create an effective, inclusive, and proactive curriculum with foresight.
- Because beliefs are often implicit, strongly held, and resistant to change, teachers must engage in conscious reflection on their beliefs (Fives & Buehl, 2016). Hence, effort should be put into assisting teachers in school based continuous professional development in reflecting on their beliefs and practices, and opportunities should be provided for teachers to experience the authentic processes of knowledge construction in order to stimulate them to rethink their belief and practice congruence.
- The Education Bureau of Addis Ababa city needs to ensure that instructional supervisory practices must be offered with most supportive and collegial manner. A worth-mentioning finding of this study is female teachers have more traditional belief while those teachers with graduate degrees were more constructivists in their belief. Consequently, the Education Bureau of Addis Ababa city needs to provide special attention to female CEE teachers by providing tailored short-term trainings and opportunities for further studies.
- Scholars (e.g., Fives et al., 2015; Levin et al., 2013) also recommend that personal reflection on one’s belief is crucial to teacher development during pre-service training. Accordingly, teacher-educators in Teacher Education Institutes should model dialogic pedagogy to create opportunities for secondary school CEE teachers to develop inquisitive mentality and reflective teaching practice during pre-service or in-service training. 

## Implication for further research

The findings of this study contribute to a better understanding of how changes aimed at improving teachers' classroom practice must take into account teachers' beliefs in conjunction with other contextual elements. The study also poses several opportunities for further  research. First, the authors recommend researchers to conduct additional studies  using the adapted Amharic version of the CLES and CTLQ instruments which we validated in this study in a different setting or level of education.

In our study, 45.8% of the variance in classroom practice was explained by teachers’ pedagogical beliefs. This is quite substantial effect. It also tells us more than half of variance in teachers’ practice can be explained by other contextual factors. Hence, we recommend researchers looking at the impact of contextual factors such as school climate and perceived teacher autonomy on teachers’ beliefs and practices. This is particularly intriguing in light of the findings of our study which revealed incongruity between constructivist belief and practice. Moreover, future studies need to incorporate qualitative element or conduct purely qualitative studies to deeply understand the ‘why’ part of CEE teachers’ belief-practice incongruity. We also recommend conducting classroom observations and including student responses to further substantiate the results.

### Limitations of the study

There are two limitations in this study. First, the data of this study was collected through self-reported responses. Hence, this study only presents the quantitative findings of a mixed design investigation of CEE teachers' pedagogical beliefs and self-reported constructivist practices in comparison with selected demographic factors. This study did not include the results of interviews or classroom observation. As a result, neither the types of actual methods/strategies used by teachers in their classrooms nor the reasons for inconsistencies in their practices were incorporated. Though we strongly believe that classroom observation and interviews could have enriched the results of the study even further, the outbreak of the Corona virus (Covid 19) has been a restraint in direct observation of classrooms.

Second, the study was conducted on CEE teachers in Addis Ababa city. Thus, we believe CEE teachers’ here are better confident to report their actual beliefs and practices which might otherwise been difficult to teachers of CEE in other sub-urban or rural parts of the country. Thus, generalizability of the findings of the study to other parts of the country needs to be cautiously considered. Apart from this, we believe that the findings of our study could be helpful in understanding the current situation of CEE teaching in secondary schools of Ethiopia.

## Data Availability

The datasets used and/or analyzed during the current study are available from the corresponding author on reasonable request.

## Abbreviations

AGFI: Adjusted goodness-of-fit index
AMOS: Analysis of moment structures
ASV: Average shared squared variance
AVE: Average variance extracted
BA/B.Ed.: Bachelor of arts/bachelor of education
CEE: Civic and ethical education
CFA: Confirmatory factor analysis
CFI: Comparative fit index
CI: Condition index
CLES: Constructivist learning environment scale
CR: Composite reliability
D-W: Durbin-Watson value
GFI: Goodness-of-fit index
KMO: Kaiser–Meyer–Olkin
MA/M.Ed.: Master of arts/masters of education
MANOVA: Multivariate analysis of variance
MaxR(H): Maximum reliability
MSV: Maximum shared squared variance
MoE: Ministry of education
OECD: Organization for economic co-operation and development
PCA: Principal component analysis
RMSEA: Root mean-square error of approximation
SRMR: Standardized root mean square residual
TLCQ: Teaching and learning conceptions questionnaire
TLI: Tucker lewis index
VIF: Variance inflation factor

## References

[CR1] Addis A.G. (2013): The role of civic and ethical education in shaping students behavior: The case of holeta secondary and preparatory school Addis Ababa university school of graduate studies institute of educational research

[CR2] Aksoy, K. (2015). What you think is not what you do in the classroom. *Procedia—Social and Behavioral Sciences*, 199, 675–683.

[CR3] Aldridge, J. M., Fraser, B. J., Taylor, P. C., & Chen, C. C. (2000). Constructivist learning environments in a cross- national study in Taiwan and Australia. *International Journal of Science Education,**22*, 37–55. 10.1080/095006900289994

[CR500] Aliusta, G. O., Özer, B., & Kan, A. (2015). The implementation of student-centred instructional strategies in schoolsin North Cyprus. *Education and Sciences*, *40*(181), 77–91.

[CR4] Althof, W., & Berkowitz, M. W. (2006). Moral education and character education: Their relationship and roles in citizenship education. *Journal of Moral Education,**35*(4), 495–518. 10.1080/03057240601012204

[CR5] Anderson, L. M., & Stillman, J. A. (2013). Student teaching’s contribution to preservice teacher development: A review of research focused on the preparation of teachers for urban and high-needs contexts. *Review of Educational Research,**83*(1), 3–69. 10.3102/0034654312468619

[CR501] Arseven, Z., Şahin, Ş., & Kılıç, A. (2016). Teachers’ adoption level of student centered education approach. *Journalof Education and Practice*, *7*(9), 133–144.

[CR6] Ashton, P. T. (2015). Historical overview and theoretical perspectives of research on teachers’ beliefs. In H. Fives & M. G. Gill (Eds.), *International handbook of research on teachers’ beliefs* (pp. 31–47). Routledge.

[CR7] Bailey, G., & Colley, H. (2015). ‘Learner-centered’ assessment policies in further education: Putting teachers’ time under pressure. *Journal of Vocational Education & Training,**67*(2), 153–168. 10.1080/13636820.2014.983956

[CR8] Banks, J. A. (2008). Diversity, group identity, and citizenship education in a global age. *Educational Researcher*, *37*(3), 129–139.

[CR9] Banks, J. A. (2007). *Educating citizens in a multicultural society* (2nd ed.). Teachers College Press.

[CR10] Baş, G., & Şentürk, C. (2019). Teaching-learning conceptions and curriculum fidelity: A relational research. *International Journal of Curriculum and Instruction,**11*(2), 163–180.

[CR11] Basturkmen, H. (2012). Review of research into the correspondence between language teachers’ stated beliefs and practices. *System,**40*(2), 282–295. 10.1016/j.system.2012.05.001

[CR12] Berger, J.-L., Girardet, C., Vaudroz, C., & Crahay, M. (2018). Teaching experience, teachers’ beliefs, and self-reported classroom management practices: A coherent network. *SAGE Open*. 10.1177/2158244017754119

[CR13] Birhanu, J. G. (2012): The Role of Civics and Ethical Education in the Development of Students’ behavior: Addis Ababa, Ethiopia

[CR14] Borg, S. (2018). Teachers’ beliefs and classroom practices. In P. Garrett & J. M. Cots (Eds.), *The Routledge handbook of language awareness* (pp. 75–91). Routledge.

[CR15] Borg, S. (2015). *Teacher cognition and language education: Research and practice*. Bloomsbury.

[CR502] Brown, T. A. (2015). *Confirmatory factor analysis for applied research* (2nd ed.). The Guilford Press.

[CR16] Buehl, M. M., & Beck, J. S. (2015). The relationship between teachers’ beliefs and teachers’ practices. In H. Fives & M. G. Gill (Eds.), *International handbook of research on teachers’ beliefs* (pp. 66–82). Routledge.

[CR17] Chan, K. W., & Elliott, R. G. (2004). Relational analysis of personal epistemology and conceptions about teaching and learning. *Teaching and Teacher Education,**20*, 817–831. 10.1016/j.tate.2004.09.002

[CR18] Chan, K., Tan, J., & Khoo, A. (2007). Pre-service teachers’ conceptions about teaching and learning: A closer look at Singapore cultural context. *Asia-Pacific Journal of Teacher Education,**35*(2), 181–195. 10.1080/13598660701268593

[CR19] Cheng, M. M. H., Chan, K. W., Tang, S. Y. F., & Cheng, A. Y. N. (2009). Pre-service teacher education student’ epistemological beliefs and their conceptions of teaching. *Teaching and Teacher Education,**25*, 319–322. 10.1016/j.sbspro.2010.03.380

[CR503] Collier, J. E. (2020). *Applied structural equation modeling using AMOS: Basic to advanced techniques*. Routledge.

[CR20] Creswell, J. W. (2012). Educational research: Planning, conducting, and evaluating quantitative and qualitative research. (4th.ed.). Boston: Pearson Education, Inc.

[CR21] Davies, I. (2000). Implementing citizenship education: Can it be done? *The School Field, XI,**3*, 91–110.

[CR22] Dawit, L. (2006). Perception of teachers and students towards civic and ethical education and its practice in selected preparatory schools of South West Shoa Zone, Unpublished. MA thesis, Addis Ababa University, Ethiopia

[CR23] Dilworth, P. P. (2008). Multicultural citizenship education. In J. Arthur, I. Davies, & C. Hahn (Eds.), *The SAGE handbook of education for citizenship and democracy* (pp. 424–437). Los Angeles, CA: SAGE.

[CR24] Endalcachew, B. (2016). Role of civics and ethical education for the development of democratic governance in Ethiopia: Achievements and challenges. *Pacific Science Review b: Humanities and Social Sciences,**2*(2016), 31–36.

[CR25] Epstein, T. (2001). Racial identity and young people’s perspectives on social education. *Theory into Practice,**40*, 42–47.

[CR26] Farrell, T., & Vos, R. (2018). Exploring the principles and practices of one teacher ofL2 speaking: The importance of reflecting on practice. *Iranian Journal of Language Teaching Research,**6*(1), 1–15.

[CR27] Farrell, T. S., & Ives, J. (2015). Exploring teacher beliefs and classroom practices through reflective practice: A case study. *Language Teaching Research,**19*(5), 594–610.

[CR28] Farrell, S. C. T., & Guz, M. (2019). ‘If i wanted to survive i had to use it’: The power of teacher beliefs on classroom practices. *The Electronic Journal for English as a Second Language,**22*(4), 1–17.

[CR29] Farrell, T., & Yang, D. M. (2017). Exploring an EAP teacher’s beliefs and practices in teaching L2 speaking: A case study. *RELC Journal,**9*, 1–14. 10.1177/0033688217730144

[CR30] Federal Democratic Republic Government of Ethiopia. (1994). *Education and training policy*. St. George Printing Press.

[CR31] Fetene, B. D. (2017). The role of civics and ethical education in shaping attitudes of students: The case of jimma college teachers education. *Global Journal of Human-Social Science: F Political Science,**17*(2), 27–58.

[CR32] Field, A. (2009). *Discovering statistics using SPSS* (3rd ed.). Sage Publications.

[CR33] Fives, H., & Buehl, M. M. (2016). Teachers’ beliefs, in the context of policy reform. *Policy Insights from the Behavioral and Brain Sciences,**3*, 114–121. 10.1177/2372732215623554

[CR34] Fives, H., & Buehl, M. M. (2012). Spring cleaning for the “messy” construct of teachers’ beliefs: What are they? Which have been examined? What can they tell us? Individual dif- ferences and cultural and contextual factorsIn K. R. Harris, S. Graham, T. Urdan, S. Graham, J. M. Royer, & M. Zeidner (Eds.), *APA educational psychology handbook* (Vol. 2, pp. 471–499). American Psychological Association.

[CR504] Fives, H., Lacatena, N., & Gerard, L. (2015). Teachers’ beliefs about teaching (and learning). In H. Fives & M. G. Gill (Eds.), *International handbook of research on teachers’ beliefs* (pp. 249–265). New York: Routledge.

[CR35] Girma, A. (2006). The Implementation of Grade 8 Civic and Ethical Education: the Case of Addis Ababa city Administration. Unpublished MA Thesis, AAU.

[CR36] Gosa, S. T. (2018). Assessment of challenges to civics and ethical education in ethiopian secondary schools. *Journal of Education and Practice,**9*(4), 16–20.

[CR37] Guerra, P. L., & Wubbena, Z. C. (2017). Teacher beliefs and classroom practices cognitive dissonance in high stakes test-influenced environments. *Issues in Teacher Education,**26*(1), 35–51.

[CR38] Hair, J. F., Jr., Black, W. C., Babin, B. J., Anderson, R. E., & Tatham, R. L. (2006). *Multivariate data analysis (6thed)*. Prentice-Hall.

[CR39] Haste, H. (2004). Constructing the citizen. *Political Psychology,**25*, 413–439. 10.1111/j.1467-9221.2004.00378.x

[CR40] Hutner, T. L., & Markman, A. B. (2016). Proposing an operational definition of science teacher beliefs. *Journal of Science Teacher Education,**27*(6), 675–691.

[CR41] Jennings, R. N. (2003). Transforming civics and citizenship education in the middle years of schooling: An exploratory of critical issues informing teachers' theories of action. Doctoral dissertation. James Cook University, Townville.

[CR505] Jones, M. G., & Leagon, M. (2014). Science teacher attitudes and beliefs. In N. G. Lederman & S. K. Abell (Eds.), *Handbook of research on science education* (Vol. II, pp. 830–847). New York, NY: Routledge.

[CR42] Kahn, J. & Wertheimer, J. (2006). The limits of political efficacy: Educating citizens for democratic society. *Political Science and Politics*. *39*(2).

[CR43] Karim, A., Reshmin, L., Kabilan, M. K., Shahed, F. H., Rahman, M. M., Singh, M. K. (2020). Understanding EFL teachers’ beliefs and practices in EFL classrooms: A phenomenological approach to the impact of teacher education program in Bangladesh. *The Qualitative Report, 25*(10), 3683–3718. 10.46743/2160-3715/2020.4272

[CR44] Kaya, Y. (2009). Democracy through learner-centered education. *International Review of Education,**55*, 21–37. 10.1007/s11159-008-9112-1

[CR45] Kerr, D. (2000). Citizenship in the national curriculum (England): Issues and challenges. *The School Field,**11*(4), 73–90.

[CR46] Kim, C., Kim, M., Lee, C., Spector, M., & DeMeester, K. (2013). Teacher beliefs and technology integration. *Teaching and Teacher Education,**29*, 76–85. 10.1016/j.tate.2012.08.005

[CR47] Kline, R. B. (2011). *Principles and practice of Structural Equation Modeling* (3rd ed.). New York: The Guilford Press.

[CR48] Knowles, R. T. (2017). Teaching who you are: Connecting teachers’ civic education ideology to instructional strategies. *Theory & Research in Social Education,**46*(1), 68–109.

[CR49] Knowles, R. T., & Castro, A. J. (2019). The implications of ideology on teachers’ beliefs regarding civic education. *Teaching and Teacher Education,**77*, 226–239.

[CR50] Lebak, K. (2015). Unpacking the complex relationship between beliefs, practice, and change related to inquiry-based instruction of one science teacher. *Journal of Science Teacher Education,**26*, 695–713.

[CR51] Lee, J., Zhang, Z., Song, H., Huang, X. (2013). Effects of epistemological and pedagogical beliefs on the instructional practices of teachers: A Chines perspective. *Australian Journal of Teacher Education*, 10.14221/ajte.2013v38n12.3

[CR52] Leem, J., & Sung, E. (2019). Teachers’ beliefs and technology acceptance concerning smart mobile devices for SMART education in South Korea. *British Journal of Educational Technology,**50*(2), 601–613. 10.1111/bjet.12612

[CR53] Levin, B.B. (2015). The development of teachers’ beliefs. In H. Fives & M. G. Gill (Eds.), *International handbook of research on teachers’ beliefs* (pp. 48–65). New York: NY: Routledge.

[CR54] Levin, B. B., He, Y., & Allen, M. H. (2013). Teacher beliefs in action: A cross-sectional, longitudinal follow-up study of teachers’ personal practical theories. *Teacher Educator,**48*(3), 1–17. 10.1080/08878730.2013.796029

[CR55] Li, L. (2013). The complexity of language teachers’ beliefs and practice: One EFL teacher’s theories. *The Language Learning Journal,**41*(2), 175–191.

[CR56] Manganelli, S., Lucidi, F., & Alivernini, F. (2015). Italian adolescents’ civic engagement and open classroom climate: The mediating role of self-efficacy. *Journal of Applied Developmental Psychology,**41*, 8–18. 10.1016/j.appdev.2015.07.001

[CR57] Mansour, N. (2013). Consistencies and Inconsistencies between Science Teachers’ Beliefs and Practices. *International Journal of Science Education,**35*(7), 1230–1275.

[CR506] Mansy, D. L. (2014). *Brain based learning: K-12 teachers' preferred methods of science instruction* (Doctoral dissertation). Retrieved from, https://search.proquest.com/pqdtglobal/docview/1650215271/fulltextPDF/E2A138BEE9EF4FABPQ/1 .

[CR58] Maurissen, L., Claes, E., & Barber, C. (2018). Deliberation in citizenship education: How the school context contributes to the development of an open classroom climate. *Social Psychology of Education*. 10.1007/s11218-018-9449-7

[CR59] Meron, T. (2006). Civic education and students of higher learning: A case in proceedings of the fourth national conference in private higher education in Ethiopia. St. Mary’s University College, Addis Ababa.

[CR60] Mhlauli, M.B. (2011). Understanding the social studies teachers’ experiences: conceptions of citizenship in Botswana, *International Journal of Scientific Research in Education*, 4(3&4), 165–180.http://hdl.handle.net/10311/1080

[CR61] Ministry of Education. (2003) Teacher education system overhaul (TESO) program, policy document, Addis Ababa.

[CR62] Ministry of Education of Ethiopia. (2009). Curriculum Framework for KG-Grade 12. Addis Ababa.

[CR63] Minstry of Education. (2018). *Ethiopian Education Development Roadmap: An Integrated executive summary (draft)*. Ministry of Education. Addis Ababa.

[CR64] Molalign M. (2012): Instructional Task Assignment and its Implementation in Civics and Ethical Education Teaching: The Case of Abyot Kirs Preparatory School in Kirkos Sub-City Addis Ababa. Addis Ababa University Office of Graduate Institute of Educational Research

[CR65] Mulugeta, Y. (2009). Building good citizenship through relevant strategies: Key remarks on the instructional process of civic and ethical education. *IER Flambeau,**16*(2), 47–69.

[CR66] Nucci, L., Creane, W., & Powers, D. W. (2015). Integrating moral and social development within middle school social studies: A social cognitive domain approach. *Journal of Moral Education,**44*(4), 479–496. 10.1080/03057240.2015.1087391

[CR67] OECD. (2009). *creating effective teaching and learning environments: First results from TALIS*. OECD Publishing.

[CR68] Pajares, M.F. (1992). Teachers’ beliefs and educational research: Cleaning up a messy construct. *Review of Educational Research*, 62, 307–332.

[CR69] Pallant, J. (2016). *SPSS survival manual: A step by step guide to data analysis using IBM SPSS*, 6th edn.

[CR70] Persson, M. (2015). Classroom climate and political learning: Findings from a Swedish panel study and comparative data. *Political Psychology,**36*(5), 587–601. 10.1111/pops.12179

[CR71] Phipps, S., & Borg, S. (2009). Exploring tensions between teachers’ grammar teaching beliefs and practices. *System,**37*(3), 380–390. 10.1016/j.system.2009.03.002

[CR72] Quintelier, E., & Hooghe, M. (2013). The relationship between political participation intentions of adolescents and a participatory democratic climate at school in 35 countries. *Oxford Review of Education,**39*, 567–589. 10.1080/03054985.2013.830097

[CR73] Riegler, A., & Quale, A. (2010). Editorial: Can radical constructivism become a mainstream endeavor? *Constructivist Foundations,**6*(1), 1–5.

[CR74] Reichert, F., Lange, D., & Chow, L. (2021). Educational beliefs matter for classroom instruction: A comparative analysis of teachers’ beliefs about the aims of civic education. *Teaching and Teacher Education,**98*(2021), 103248. 10.1016/j.tate.2020.1032480742-051X/

[CR75] Reichert, F., & Torney-purta, J. (2019). A cross-national comparison of teachers‟ beliefs about the aims of civic education in 12 countries: A person-centered analysis. *Teaching and Teacher Education,**77*, 112–125.

[CR76] Rodriguez, A., & Magill, K. R. (2016). Diversity, neoliberalism, and teacher education. *International Journal of Progressive Education,**12*(3), 7–23.

[CR77] Rubie-Davies, C. (2015). Teachers’ instructional beliefs and the classroom climate: Connections and conundrums. In H. Fives & M. G. Gill (Eds.), *International handbook of research on teachers’ beliefs* (pp. 266–283). Routledge.

[CR78] Schuitema, J., Veugelers, W., & Rijlaarsdam, ten Dam, G.,. (2009). Two instructional designs for dialogic citizenship education: an effect study. *British Journal of Educational Psychology,**79*, 439–461. 10.1348/978185408X393852 19224680 10.1348/978185408X393852

[CR79] Semela, T., Bohl, T., & Kleinknecht, M. (2013). Civic education in Ethiopian schools: Adopted paradigms, instructional technology, and democratic citizenship in a multicultural context. *International Journal of Educational Development,**33*(2), 156–164. 10.1016/j.ijedudev.2012.03.003

[CR80] Şentürk, C. & Zeybek, G. (2019). Teaching-learning conceptions and pedagogical competence perceptions of teachers : a correlational research. *Research in Pedagogy*, *9*(1), 65–80. 10.17810/2015.92

[CR81] Sing, C., & c., & Khine, M.S. (2008). Assessing the epistemological and pedagogical beliefs among pre- service teachers in Singapore. In M. S. Khine (Ed.), *Knowing, knowledge and beliefs: Epistemological studies across diverse cultures* (pp. 287–303). Springer.

[CR82] Skott, J. (2015). The promises, problems, and prospects of research on teachers’ beliefs. In H. Fives & M. G. Gill (Eds.), *International handbook of research on teacher’s beliefs* (pp. 13–30). Routledge.

[CR83] Tabachnick, B. G., & Fidell, L. S. (2013). *Using multivariate statistics* (6th ed.). Pearson Education.

[CR84] Tabachnick, B. G., & Fidell, L. S. (2007). *Using multivariate statistics* (5th ed.). Pearson.

[CR85] Taylor, P. C., Fraser, B. J., & Fisher, D. L. (1997). Monitoring constructivist classroom learning environments. *International Journal of Educational Research,**27*, 293–302. 10.1016/S0883-0355(97)90011-2

[CR86] Thibaut, L., Knipprath, H., Dehaene, W., & Depaepe, F. (2018). The influence of teachers’ attitudes and school context on instructional practices in integrated STEM education. *Teaching and Teacher Education,**71*(1), 190–205.

[CR87] Thomas, B. I., Comfort, O. O., & Adams, O. E. (2013). Teacher educators’ conception of teaching and learning in teacher education institutions. *International Journal of Research Studies in Education.,**2*(2), 43–52.

[CR507] Wachob, D. A. (2012). *Public school teacher knowledge, perception, and implementation of brain-based learning practices* (Doctoral Dissertation). Retrieved from, http://www.proquest.com/pqdtglobal/docview/1240671335/fulltextPDF/F07FEBAA21F7443EPQ/1.

[CR88] Waltainfo .(2017). Civics education far behind from meeting set goals: Study. April 20, Retrieved August 4, 2021, from Walta Information Center website:. https://www.waltainfo.com/news/national/detail?cid=29139&locale=en&locale=en

[CR508] Wang, P. (2016). Teachers’ implementation of constructivist teaching: Does career motivation make a difference? Theses and Dissertations (All). 1396. http://knowledge.library.iup.edu/etd/1396

[CR89] Wong, S. S., & Luft, J. A. (2015). Secondary science teachers’ beliefs and persistence: A longitudinal mixed-methods study. *Journal of Science Teacher Education,**26*, 619–645.

[CR90] Yamada S. (2014). *Domesticating Democracy*? In: Williams J.H. (eds) (Re) Constructing Memory. Sense Publishers, Rotterdam. 10.1007/978-94-6209-656-1_3

[CR91] Zhang, F., & Liu, Y. (2014). A study of secondary school English teachers’ beliefs in the context of curriculum reform in China. *Language Teaching Research.,**18*(2), 187–204. 10.1177/1362168813505940

